# ‘All in a box’ a concept for optimizing microbiological diagnostic sampling in prosthetic joint infections

**DOI:** 10.1186/1756-0500-7-418

**Published:** 2014-07-03

**Authors:** Lone Heimann Larsen, Yijuan Xu, Ole Simonsen, Christian Pedersen, Henrik C Schønheyder, Trine Rolighed Thomsen

**Affiliations:** 1 Department of Clinical Microbiology, Aalborg University Hospital, Aalborg, Denmark; 2 Center for Microbial Communities, Institute for Biotechnology, Chemistry and Environmental Engineering, Aalborg University, Aalborg, Denmark; 3 Section for Medical Biotechnology, Life Science Division, Danish Technology Institute, Aarhus, Denmark; 4 Department of Orthopaedic Surgery, Aalborg University Hospital, Aalborg, Denmark; 5 Department of Clinical Medicine, Aalborg University, Aalborg, Denmark

**Keywords:** Prosthesis, Infections, Specimen handling, Specimen types, Transport media

## Abstract

**Background:**

Accurate microbial diagnosis is crucial for effective management of prosthetic joint infections. Culturing of multiple intraoperative tissue samples has increased diagnostic accuracy, but new preparatory techniques and molecular methods hold promise of further improvement. The increased complexity of sampling is, however, a tough challenge for surgeons and assistants in the operation theatre, and therefore we devised and tested a new concept of pre-packed boxes with a complete assortment of swabs, vials and additional tools needed in the operating theatre for non-standard samples during a clinical study of prosthetic joint infections.

**Findings:**

The protocol for the clinical study required triplicate samples of joint fluid, periprosthetic tissue, bone tissue, and swabs from the surface of the prosthesis. Separate boxes were prepared for percutaneous joint puncture and surgical revision; the latter included containers for prosthetic components or the entire prosthesis. During a 2-year project period 164 boxes were used by the surgeons, 98 of which contained a complete set of samples. In all, 1508 (89%) of 1685 scheduled samples were received.

**Conclusion:**

With this concept a high level of completeness of sample sets was achieved and thus secured a valid basis for evaluation of new diagnostics. Although enthusiasm for the project may have been a contributing factor, the extended project period suggests that the ‘All in a box’ concept is equally applicable in routine clinical settings with standardized but complex diagnostic sampling.

## Findings

### Background

The microbiological diagnosis plays a crucial role in the effective management of patients with suspected prosthetic joint infection (PJIs) [1]. Diagnostic procedures include percutaneous aspiration of joint fluid as well as revision surgery with retention or removal of prosthetic elements. Chronic foreign body-related infections pose a special challenge because of the diversity of microorganisms involved and their adaption to a subdued lifestyle associated with formation of biofilms. Culturing of multiple samples has been shown to increase diagnostic accuracy, and there is growing evidence to support the utility of new preparatory techniques and molecular methods [2-4].

As a direct consequence of this development the number and types of samples wanted from the surgical field are increasing, and the sampling procedure thereby becomes more cumbersome for the surgeon. Even with assistance from a skilled nurse on the floor of the operating theatre important samples can be missed or deposited in an unsuitable transport medium, and the diagnostic accuracy can thereby be compromised [2,4-6].

Within an ongoing research project comprising patients with a painful prosthetic joint (‘Prosthesis: Related Infection and Pain’ (PRIS), http://www.joint-prosthesis-infection-pain.dk) we have addressed this issue by designing pre-packed boxes containing disposable scalpels and forceps, swaps, transport vials, and labels needed for sampling during the surgical procedure. Additional boxes were made available for samples of synovial fluid obtained by percutaneous joint aspiration. Our primary aim was to overcome the variation in sampling technique within and between surgical teams and across difference hospitals, which otherwise might affect the validity of our clinical study. Our belief was that streamlining sampling procedures would maximize the completeness of sample sets. We here present the results from a 2-year project period.

### All in a box

We applied the ‘All in a box’ concept to two surgical procedures and report the completeness of sampling within a prospective cohort of patients undergoing revision surgery.

The concept was developed jointly by orthopaedic surgeons, molecular biologists, and clinical microbiologists within the framework of the PRIS project. The project was approved by the Regional Committee on Health Research Ethics (June 2011; ref. no. N-20110022). Informed oral and written consent was obtained from each patient.

The sample repertoire was supplementary to five intraoperative soft tissue biopsies obtained according to the Kamme and Lindberg principle [7]. For revisions the non-standard samples comprised joint fluid, intraoperative soft tissue and bone biopsies, swabs from the surface of the prosthesis *in situ*, and prosthetic components or the entire prosthesis. Diagnostic methods included bacteriological culture, 16S *rDNA* gene amplification followed by amplicon sequencing, and fluorescence *in situ* hybridization (FISH). Thus, samples were obtained in triplicate except for the prosthesis itself or prosthetic components. Each sample was handled separately with disposable utensils in order to minimize cross-contamination [7,8] and thus allow valid comparison of different sample types and analyses.

**Figure 1:**
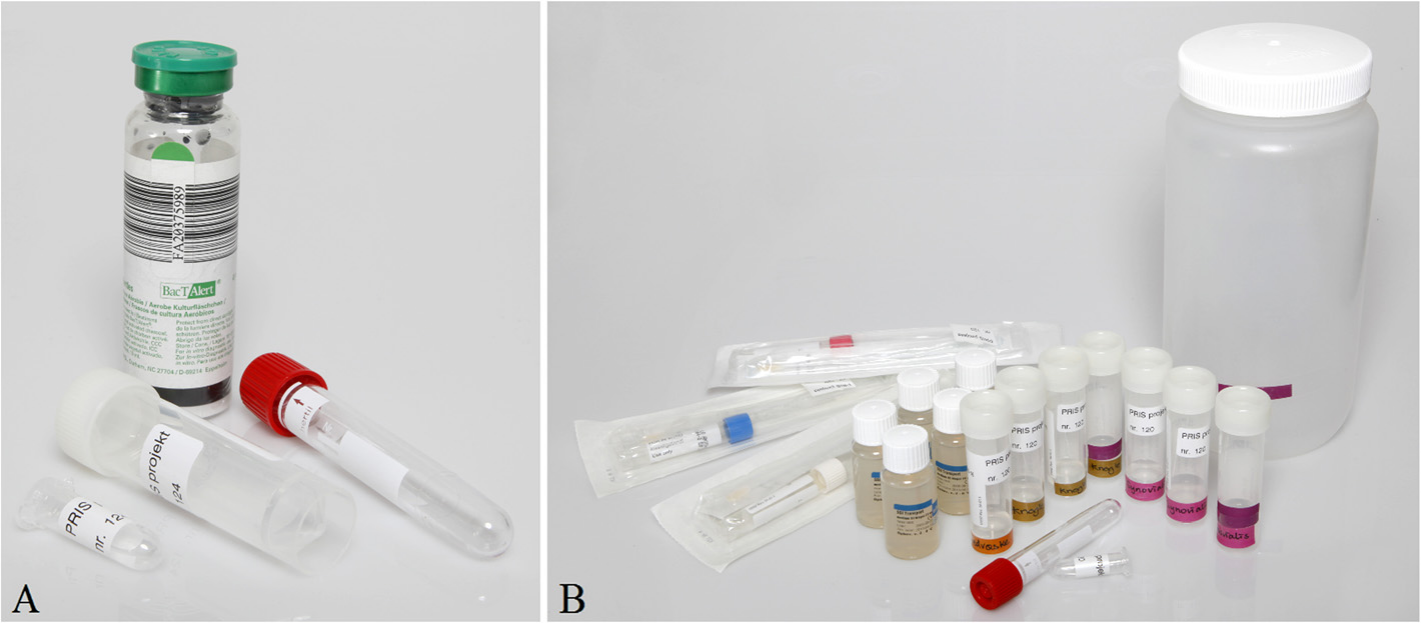
**Sample boxes for joint puncture (A) and revision surgery (B). A**: Joint fluid is both inoculated directly into a blood culture flask (BacT/Alert, bioMérieux, Marcy l’Etoil, France) and submitted for extensive culture examination and molecular diagnostics. **B**: Sample tubes are colour coded in the revision surgery box in order to assist the operation staff in achieving complete sample sets. Sample tubes had a broad neck in order to facilitate the deposition of the sample.

The two types of boxes are presented in Figure 1 and Table 1. The sample collecting kit for revision surgery consisted of scalpels and forceps, and a special needle for a bone biopsy (Vertebroplasty Needle, Synthes, West Chester PA, USA). Sample tubes were colour coded according to sample type. For collection of biopsies, tubes with a broad neck were chosen to facilitate handling in the operating theatre as well as in the laboratories. A sterile container of an appropriate size for the prosthetic component was included for revision surgery. The only item not included in the pre-packed boxes was a blood culture vial for synovial fluid due to its limited shelf life.

**Table 1:** Boxes’ design and transport media

	**Specimen type**	**Bacteriological culture**	***16S rDNA *** **sequencing**	**FISH**
**Joint puncture**	Synovial fluid	15 mL tube (empty)*	Tube B	Tube C
		Blood culture vial		
**Revision surgery**	Synovial fluid	15 mL tube (empty)	Tube B	Tube C
	Soft tissue	Tube A	Tube D	Tube C
		Vials with Stuart transport medium (x5)*		
	Bone biopsies	Tube A	Tube D	Tube C
	Swabs from prosthesis***	Tube A	Tube D	Tube C
		(ESwab)		
	Prosthesis (components or in toto)	Empty container**	Tube B**	Tube C**

Tube A: Modified Amies medium (Copan). Tube B: 2 mL tube with 60% glycerol in DEPC water, targeting a final concentration ≥10% glycerol including sample; estimated final concentration ~15-20%. Tube C: CyMol® (Copan). Tube D: Modified Amies medium with 20% glycerol (Copan). The transport media were 1] modified Amies medium for direct culture, 2] CyMol® for the preservation of nucleic acids for FISH, and 3] modified Amies medium with 20% glycerol (estimated final glycerol content ~10% incl. sample) for storage of whole bacterial cells at -80°C for subsequent molecular analyses (Copan). Stuart medium (SSI Diagnostica, Copenhagen Denmark) was used for biopsies of periprosthetic soft tissue obtained according to Kamme and Lindberg [7] and handled accordingly since the 1980’s.

*Samples taken routinely during surgical revision.

**The processing of prosthetic components took place in the laboratory (summarized in [2]). ***Swabs used to prosthetic scraping *in situ* for culture and 16S *rDNA* gene sequencing were CLASSIQSwab and for FISH (CyMol®) a FLOQSwab (Copan).

From the surgical theatre the boxes were transported at ambient temperature to the Department of Clinical Microbiology and processed within 24 h. Most samples were processed within 2 h after removal of the prosthesis, whereas samples from acute surgery undertaken out of hours were kept at 4°C overnight except for the blood culture vial that was held at room temperature. When delivered to the lab, samples for molecular analysis were subjected to vigorous agitation (vortexing for 30 sec) and stored immediately at -80°C until batch wise processing and analysis. For microbiological culture components of the prosthesis (covered with PBS-buffer, pH7.4) were vortexed and sonicated (summarised in [2]). Bone biopsies were treated similarly before culturing. The joint fluid, tissue biopsies, and the prosthesis swab were cultured without pre-processing. All sample types were cultured aerobically and anaerobically for 14 days with subcultivation from enrichment broth after 6 days for positive samples and after 10 days for negative samples.

All surgeons undertaking revision surgery were informed about the box design and agreed to the concept. The implementation benefitted further from liaison with the nurses assisting in the operating theatre. Of note, joint punctures took place in both ambulatory and in-hospital settings, and they were less rigidly standardized compared with revisions.

### ‘Proof of concept’

The scheduled number of samples was four for percutaneous joint aspiration (box A) and 13 for revision surgery (box B) (Table 1). From December 2011 through February 2014 we obtained 98 boxes with a complete sample set out of 164 consecutive boxes (box A: 25/42 (60%); box B: 73/122 (60%)). In all, 1508 of 1685 scheduled samples were obtained (overall completeness 89%). The main reasons for missing samples were deviations from the pre-planned surgical procedure for clinical reasons or absence of a trained assistant. In 8 cases the sample set in box B was incomplete as a consequence of acute surgery (69 of 104 scheduled samples (66%)).

### Experience and perspective

We find the ‘All in a box’ to be a promising logistic concept for obtaining multiple samples as part of surgical procedures. The concept may be applicable not only to the diagnosis of PJIs but also to other diagnostic procedures and would be well suited especially in circumstances where limited amounts of sample material must be shared between several diagnostic tests and the use of a correct transport medium plays an important role for the performance of the diagnostic test. An obvious addition to the different microbiological tests in this clinical study would be tissue samples for histopathology. Despite the complex intraoperative sampling procedure the ‘All in a box’ concept provided an overall completeness around 90% in a research project involving several orthopaedic surgeons, numerous nurses, and different hospital premises.

The concept should also be applicable to other complex sampling procedures utilizing a standardized panel of diagnostic sample types and thus has a potential for time saving and optimization in different diagnostic settings. Although we ascribe the high level of completeness in our study to the ‘All in a box’ concept, it must be acknowledged that enthusiasm surrounding the research project may also have been involved. Still, the positive attitude often withers when procedures are complicated and involve many surgeons and nurses, but it was our impression that the box logistics helped to maintain the spirit in this case. A drawback to the concept was the time consumed by the meticulous preparation of the boxes, a task which can be managed within the framework of a scientific project, but may be difficult to tackle on a routine basis in hospitals and clinics. We estimate the cost of materials for box A to be 130 €, and the full diagnostic work-up of samples in this box may amount to 1075 € including extensive 16S *rDNA* sequencing. Implementation of only the most effective diagnostic modalities may help to decrease these costs. Moreover, the concept could be of interest to providers of diagnostic utensils and could also be instrumental in implementing standardized sampling procedures eventually based on international guidelines.

## Competing interests

The authors declare that they have no competing interest.

The transport medium ‘Modified Amies media with 20% glycerol’ was developed in cooperation between the authors and Copan Italia S.P.A. (Brescia, Italy). All transport media were provided by Copan, if not otherwise indicated. Copan did not influence on the manuscript and the authors have no financial interests to declare.

## Authors’ contributions

OS, TRT and HCS designed and developed the first versions of the boxes. After inputs from YX and CP the first pilot study was conducted with OS and CP as surgeons [3]. LHL, YX, TRT, HCS and OS optimized the boxes for the clinical study. LHL and YX were responsible for the overall management of the boxes. LHL prepared the first draft of the manuscript and all authors read and approved the final manuscript.
